# Technique for areolar reduction areolar-sparing mastectomy

**DOI:** 10.1093/jscr/rjae824

**Published:** 2024-12-27

**Authors:** Zachary Lawrence, Joel C Stroman, Heather Karu

**Affiliations:** Department of Surgery, University of South Dakota Sanford School of Medicine, 1400 W 22nd St, Sioux Falls, SD 57105, United States; University of South Dakota Sanford School of Medicine, 414 E. Clark Street, Vermillion, SD 57069, United States; Department of Plastic and Reconstructive Surgery, University of South Dakota Sanford School of Medicine, 1500 W. 22nd St. Suite 101, Sioux Falls, SD 57105, United States

**Keywords:** areola reduction, breast surgery, areolar-sparing mastectomy

## Abstract

Achieving satisfactory nipple esthetics following skin-sparing or nipple-sparing mastectomy is an important element of ensuring positive patient outcomes. Several techniques used to reconstruct the nipple–areolar complex have been described in previous literature and have had success in securing good cosmetic outcomes. For patients with macrothelia, surgeons may employ a number of options in reducing nipple size. Existing studies have shown common preferences among individuals surveyed regarding breast cosmesis, specifically related to the nipples and areolae. However, for individuals with disproportionately large areolae following areolar-sparing mastectomy, there is scant literature to suggest methods of reducing the size of the areolae, and current practice appears to be based upon fixed diameters of areolar sizers. In this technical report, we describe a suture-only technique that successfully reduced areola diameter and recreated the appearance of a nipple with a small central projection following areolar-sparing mastectomy.

## Introduction

Over the past four decades, many methods of varying complexity have been created in pursuit of finding an ideal method for closing breast wounds after surgical procedures that involve removal of the nipple or areolae [1, 2]. While this is straightforward in patients with a common-sized areola, this becomes more difficult to perform areolar-sparing mastectomies in patients with large areolae. The degree of difficulty in achieving a cosmetically favorable result after these types of operations has prompted academic investigation well beyond the operative arena into areas such as 3D printing [3].

Given the increased number of patients undergoing nipple-sparing and areolar­sparing mastectomies in recent years, a reliable method is needed for surgeons at institutions where advanced technologies may not be available, such as rural hospitals [4]. In this technical report, we describe a novel, easy-to-perform method for areolar reduction areolar-sparing mastectomy requiring only Vicryl and nylon suture.

## Case presentation

The patient is a 48-year-old female who presented to our clinic from a rural town in 2022 to discuss options for breast reconstruction following bilateral mastectomy in the setting of invasive lobular carcinoma of the right breast. She was determined to be a candidate for nipple-sparing mastectomy; however, she was unhappy with the size of her areolae and expressed a strong desire for concomitant removal of ductal tissue. The patient ultimately elected for bilateral areolar-sparing mastectomy with direct-to-implant reconstruction and a plan was made to reduce the size of her areolae and then close the remaining areolar tissue in a centripetal fashion. Though there was no intention to reconstruct her nipple formally, we believed this type of closure would be a satisfactory alternative as it would provide layered closure of the surgical wounds and provide a small amount of projection centrally in the position of the nipple. Three weeks after her initial consultation, she underwent direct-to-implant reconstruction with areolar reduction centripetal areolar closure following the performance of an initial bilateral nipple-sparing mastectomy by the breast surgeon.

## Methods

**Figure 1:**
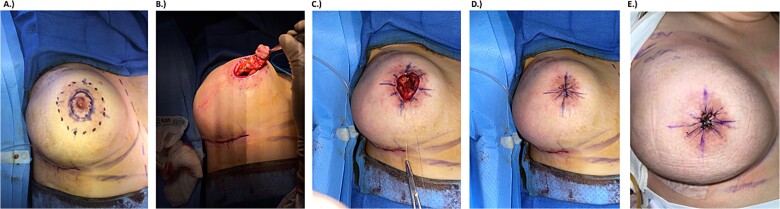
Intraoperative photodocumentation for surgical technique and outcome. (a) Preoperative marking. Solid line shows the portion to be removed, dotted line marks the areolar border. (b) Post-mastectomy removal of nipple. (c) Four-point purse string closure for deep tissue. (d) Eight-point purse string closure for superficial tissue. (e) One month follow-up photo.

First, the outside of the areola was marked, and then another marking was made circumferentially 2 cm within the outer areolar border (Fig. 1). The central area including the nipple and excess areola was then excised down to the level of the sizer, ensuring removal of the nipple and underlying ductal tissue while leaving a cuff of dermis superficially (Fig. 1).

Areolar closure was then carried out in four layers. The deepest portion of the flap was closed with 2–0 Vicryl suture using a four-point purse string technique, which was repeated to close the deep dermis (Fig. 1). The superficial dermis was then approximated with an 8-point 2–0 Vicryl purse string suture followed by an 8-point purse string with 3–0 nylon at the areolar surface, drawing the wound edges together centripetally (Fig. 1). Interrupted 4–0 nylon sutures were then placed on each of the eight tissue segments for further fine­tuning and support. A smooth, round, high-profile implant was wrapped in acellular dermal matrix for soft tissue reinforcement and secured in the prepectoral space along with a surgical drain. The same process was repeated for the right breast. Remaining areolar tissue was well perfused bilaterally at the conclusion of the operation.

The patient was seen for follow-up at 2, 4, and 8 weeks postoperatively. Surgical drains were removed after 16 days. Nylon sutures were removed after 61 days. Areolar diameter was symmetric at 8 weeks. No complications were reported.

## Discussion

There are myriad approaches to achieving acceptable nipple appearance following skin-sparing or nipple-sparing mastectomy, yet none have emerged as the gold standard [5]. In the setting of skin-sparing mastectomies requiring nipple–areolar complex (NAC) reconstruction, several iterations of flap-based techniques have previously been described with varying results. A recent paper describing a new flap-based technique reported reductions in rates of nipple retraction and projection loss that often complicate NAC reconstruction [6].

Other methods include the use of tissue grafting, nipple sharing, or 3D tattoos, with tattooing typically completed sometime after the initial operation [5, 7, 8]. However, a combined single-stage technique has been described [9]. Through complex tissue engineering and grafting, some authors have found success in reconstructing the NAC using cadaveric tissue [10]. Patient satisfaction with NAC reconstruction is typically based upon projection, texture, and color of the nipple [9].

Though literature on the subject is sparse, some authors have attempted to determine ideal nipple dimensions following nipple-sparing mastectomy.

Results from a recently published survey indicate an areola width to breast width ratio of 2–12 or 3–12 are considered the ideal proportions among a mixed sample [11]. Areola sizer diameters of 38 and 45 mm have influenced the range generally accepted by breast surgeons, though surgeon preference intraoperatively and differences between patients lead to some variation [11, 12]. Several methods of nipple reduction in the setting of macrothelia have been described, though none are noted to specifically address large areolae [13].

While each of the aforementioned methods can be useful strategies in achieving good nipple cosmesis in different settings, many are highly technical, require advanced equipment, or do not address the areolae, which renders them impractical to surgeons in rural areas with fewer resources faced with this issue.

This patient with invasive lobular carcinoma underwent bilateral areolar-sparing mastectomy, after which her areolae were down-sized using a combination of absorbable and nonabsorbable suture for four-layer purse string closure with a simple interrupted technique to create the appearance of a small central papilla. Her nipples were symmetric at the 8-week follow-up, and she was satisfied with her results. Though this represents a single application of the method, we hope the success of this simple technique may prove useful to other surgeons and benefit patients requiring areola reduction in the rural setting.
